# Effectiveness of the live attenuated and the inactivated influenza vaccine in two-year-olds – a nationwide cohort study Finland, influenza season 2015/16

**DOI:** 10.2807/1560-7917.ES.2016.21.38.30346

**Published:** 2016-09-22

**Authors:** Hanna Nohynek, Ulrike Baum, Ritva Syrjänen, Niina Ikonen, Jonas Sundman, Jukka Jokinen

**Affiliations:** 1 Vaccine Programme Unit, Department of Health Protection, National Institute for Health and Welfare, Finland; 2 Impact Assessment Unit, Department of Health Protection, National Institute for Health and Welfare, Finland; 3 Viral Infections Unit, Department of Infectious Diseases, National Institute for Health and Welfare, Finland

**Keywords:** influenza, vaccines, immunisation, live attenuated vaccine, inactivated vaccine, children

## Abstract

Although widely recommended, influenza vaccination of children is part of the national vaccination programme only in few countries. In addition to Canada and the United States (US), in Europe Finland and the United Kingdom have introduced live attenuated influenza vaccine (LAIV) for healthy children in their programmes. On 22 June 2016, the US Advisory Committee on Immunizations Practices, voted against further use of LAIV due to no observed vaccine effectiveness (VE) over three consecutive influenza seasons (2013/14 to 2015/16). We summarise the results of a nationwide, register-based cohort study (N=55,258 of whom 8,086 received LAIV and 4,297 TIV); all outcome (laboratory-confirmed influenza), exposure (vaccination) and confounding variable data were retrieved from four computerised national health registers, which were linked via a unique personal identity code assigned to all permanent Finnish residents regardless of nationality. Our study provides evidence of moderate effectiveness against any laboratory-confirmed influenza of the quadrivalent LAIV vaccine (VE: 51%; 95% confidence interval (CI): 28–66%) as well as the inactivated trivalent vaccine (VE: 61%; 95% CI: 31–78%) among two-year-olds during the influenza season 2015/16 in Finland. Based on these data, Finland will continue using LAIV for young children in its National Immunisation Programme this coming influenza season.

## Introduction

Influenza causes mild to severe symptoms among one in three young children. Vaccination is considered the best available intervention to prevent influenza in children and its spread from children to other age groups reducing the disease burden in the entire population [1]. Many European countries recommend to vaccinate the elderly, medical risk groups and healthcare workers but only nine countries recommend vaccination of healthy children, i.e. Austria, Estonia, Finland, Latvia, Malta, Poland, Slovakia, Slovenia, and the United Kingdom (UK) [2].

Since 2007, influenza vaccine has been given free of charge to all children aged 6 to 35 months as part of the National Vaccination Programme of Finland (NVP) [3], following a formal cost effectiveness analysis [4] requested by the National Immunization Technical Advisory Group and favourable decision by the government. For young healthy children and those above three but under nine years of age with medical risk conditions, the recommended schedule has included two doses for those vaccinated for the first time ever and one dose if they were already vaccinated during previous seasons.

Different types of influenza vaccines have been available for large scale use since early 1970s. Inactivated influenza vaccines have been commonly used. The live attenuated influenza vaccine (LAIV) was developed already in the 1960s but it has been available for large scale use in the United States (US) since 2003 (FluMist) and in Europe since 2011 (Fluenz). Prior to season 2015/2016, in Europe, only the UK had introduced LAIV for healthy children in their programme.

During the influenza season 2015/16, for the first time in Finland, two-year-olds (i.e. children aged 24 to 35 months) were offered either one or two doses of trivalent inactivated influenza vaccine (TIV; Vaxigrip) or one dose of LAIV (FluenzTetra). No preference for either was made in the national recommendation. Both vaccines were scheduled to be given in November and December 2015, although TIV could also be used from 6 January 2016 onwards after LAIV doses available in NVP had expired.

On 22 June 2016, the US Advisory Committee of Immunization Practices (ACIP) discussed the effectiveness of LAIV given to children from 2 to 17 years of age over three consecutive seasons in the US. Due to no observed vaccine effectiveness using the test negative design methodology, the ACIP voted against the use of LAIV in children during the coming season 2016/17 [5]. However, mid-season data from both Finland and the UK made available to the ACIP via CDC demonstrated reasonable effectiveness of the LAIV vaccine produced in the same plant [6,7].

As part of its statutory tasks, the Finnish National Institute for Health and Welfare (THL) is obliged to monitor the effectiveness and safety of vaccines used, in order to measure the impact of the NVP, and to give evidence-based vaccination recommendations [3]. Finland recently established a nationwide, computerised, real-time vaccination register (NVR) [8]. Linking NVR with disease register data in real time allows comprehensive effectiveness studies in timely manner. We present the end-of-season estimate of the influenza vaccine effectiveness (VE) among all two-year-old children residing permanently in Finland during the influenza season 2015/16 using national register data.

## Methods

### Study design and follow-up period

This nationwide register-based cohort study retrospectively assessed influenza VE in two-year-old children, i.e. the birth cohort of 2013, during the influenza season 2015/16, defined as lasting from week 40 (28 September 2015) to week 20 (22 May 2016). All outcome, exposure and confounding variable data were retrieved from four computerised national health registers maintained by THL, which were linked via a unique personal identity code assigned to all permanent Finnish residents regardless of nationality.

### Study population

The study population, i.e. the birth cohort of 2013, was defined based on the Finnish Population Register, which contains an up-to-date information of all permanent residents in Finland. 

### Exposure

Vaccination status was defined by the NVR, which contains individual-level vaccination records comprising the vaccinee’s personal identity code, the administered vaccine (including brand name) and the date of vaccination. The NVR covers records of vaccinations given from 2009 onwards in public primary healthcare, which is responsible for the delivery of the NVP. However, small regional and temporal information gaps are assumed, mainly due to data dispatch problems [8]. Every individual within the study population and with at least one recorded influenza vaccination in the NVR in 2015/16 was considered vaccinated since the day of vaccination. For purposes of sensitivity analysis, children were also considered vaccinated only after a two-week-period following vaccination allowing them to develop a sufficiently protective immunity. Consecutive vaccinations within the same season are rare among two-year-olds, and observed in less than 1% of those vaccinated. They were not considered in the analysis.

### Outcome

The outcome of interest was any laboratory-confirmed influenza (LCI) registered in the National Infectious Disease Register (NIDR). The NIDR covers nationwide data about LCI cases, diagnosed in both public and private primary and secondary care. No universal recommendation exists when a suspected case should be tested for influenza. In Finland, influenza suspected patients are tested for influenza by RT-PCR, multiplex RT-PCR, culture and/or antigen detection and all influenza-positive cases from all laboratories are reported to the NIDR, where the patient’s personal identity code, the influenza type, and the date of laboratory confirmation is recorded. In this report, LCI was defined as influenza finding in RT-PCR, multiplex RT-PCR, culture and/or antigen detection test, and further stratified to LCI type A and LCI type B.

### Confounders

In order to control for potential confounders, several variables describing the characteristics of the study population were included in the analysis. Background information was collected from the Finnish National Medical Birth Register (NMBR), which contains data about the status of the child and the mother at the time of child’s birth [9]. The following 12 categorical variables (levels given in Table 1) were considered in the analysis: mother’s age at birth in years (<20, 20–24, 25–29, 30–34, 35–39, ≥40), socio-economic status ( based on mother´s profession), marital status and smoking behaviour, as well as child’s birth weight in grams (<1,500, 1,500-2,499, ≥2,500), gestational age at birth in weeks, number of siblings at birth, month of birth (January–June, July–December) as indicator for the eligibility to previous seasonal influenza vaccinations, sex, nationality, place of residence, and BCG (Bacillus Calmette–Guérin) vaccination status.

**Table 1:** Baseline characteristics of two-year-old children by seasonal influenza vaccination status, Finland, influenza season 2015/16 (n= 55,258)

	Not vaccinated (N=42,875)	LAIV vaccinated (N=8,086)	TIV vaccinated (N=4,297)	p-value^a^
**Mother’s age at birth^b^**				
**Years**	30 (5.3)	31 (5.0)	31 (5.0)	<0.001
**Socio-economic status based on mother’s occupation^c,e^**				
Higher white-collar workers	8,596 (20.0)	2,158 (26.7)	1,145 (26.6)	<0.001
Lower white-collar workers	18,375 (42.9)	3,329 (41.2)	1,760 (41.0)	
Blue-collar workers	7,069 (16.5)	934 (11.6)	516 (12.0)	
Others	8,835 (20.6)	1,665 (20.6)	876 (20.4)	
**Mother’s marital status^d^**				
Single or divorced	4,202 (9.8)	620 (7.7)	334 (7.8)	<0.001
Cohabiting	14,830 (34.6)	2,408 (29.8)	1,210 (28.2)	
Married	23,843 (55.6)	5,058 (62.6)	2,753 (64.1)	
**Mother’s smoking behaviour^d^**				
No	35,303 (82.3)	7,284 (90.1)	3,867 (90.0)	<0.001
Quitted during first trimester	3,232 (7.5)	427 (5.3)	210 (4.9)	
Continued after first trimester	4,340 (10.1)	375 (4.6)	220 (5.1)	
**Birth weight^b,d^**				
Grams	3,514 (541.8)	3,470 (579.7)	3,459 (595.1)	<0.001
**Gestational age at birth^d^**				
<28 weeks	68 (0.2)	35 (0.4)	30 (0.7)	<0.001
≥28 and <37 weeks	4,173 (9.7)	903 (11.2)	504 (11.7)	
≥37 weeks	38,634 (90.1)	7,148 (88.4)	3,763 (87.6)	
**Number of siblings at birth^c^**				
0	16,156 (37.7)	4,057 (50.2)	1,830 (42.6)	<0.001
1	15,116 (35.3)	2,465 (30.5)	1,509 (35.1)	
>1	11,603 (27.1)	1,564 (19.3)	958 (22.3)	
**Month of birth^c^**				
January–June	22,169 (51.7)	3,424 (42.3)	1,967 (45.8)	<0.001
July–December	20,706 (48.3)	4,662 (57.7)	2,330 (54.2)	
**Sex^c^**				
Male	21,870 (51.0)	4,225 (52.3)	2,302 (53.6)	0.001
Female	21,005 (49.0)	3,861 (47.7)	1,995 (46.4)	
**Nationality^c^**				
Finnish	39,483 (92.1)	7,682 (95.0)	4,013 (93.4)	<0.001
Non-Finnish	3,392 (7.9)	404 (5.0)	284 (6.6)	
**Place of residence^c^**				
Urban	29,709 (69.3)	6,220 (76.9)	3,368 (78.4)	<0.001
Semi-urban	7,713 (18.0)	1,125 (13.9)	517 (12.0)	
Rural	5,453 (12.7)	741 (9.2)	412 (9.6)	
**BCG vaccination status^c^**				
Not vaccinated	39,403 (91.9)	7,618 (94.2)	3,988 (92.8)	<0.001
Vaccinated	3,472 (8.1)	468 (5.8)	309 (7.2)	
**Presence of underlying chronic conditions^c^**				
No	37,734 (88.0)	7,032 (87.0)	3,510 (81.7)	<0.001
Yes	5,141 (12.0)	1,054 (13.0)	787 (18.3)	
**Presence of an acute disease between week 14–39, 2015^c^**				
No	39,766 (92.7)	7,354 (90.9)	3,791 (88.2)	<0.001
Yes	3,109 (7.3)	732 (9.1)	506 (11.8)	
**SIV vaccination status in2013/14 and 2014/15^c,f^**				
Not vaccinated	38,288 (89.3)	3,470 (42.9)	1,386 (32.3)	<0.001
Vaccinated	4,587 (10.7)	4,616 (57.1)	2,911 (67.7)	

BCG: Bacillus Calmette–Guérin vaccine: LAIV: live attenuated influenza vaccine; TIV: trivalent inactivated influenza vaccine; SIV: seasonal influenza vaccine.

^a^ One-way analysis of variance for continuous and chi-squared test of independence for categorical variables.

^b^ Mean (standard deviation).

^c^ Absolute frequency (relative frequency in %). Because of rounding, percentages may not total 100.

^d^ Proportion of data imputed by hot deck imputation: <0.2%.

^e^ Proportion of data imputed by hot deck imputation: 31.5%.

^f^ Vaccinated group contains those vaccinated either in the 2013/14, 2014/15 season or both.

Acute and chronic diagnoses made in hospitals were extracted from the National Register of Health Care (NRHC), which covers diagnosis information of all outpatient and inpatient healthcare provided in Finnish hospitals [10]. The following three acute diseases diagnosed within 6 months before the vaccination campaign (weeks 14–39 in 2015) and 13 chronic disease entities from birth until the end of 2015 were selected based on their International Statistical Classification of Diseases and Related Health Problems tenth Revision (ICD-10) codes [11]: acute bacterial and viral infections (A30–A49, A85–A89), acute diseases of the middle ear (H65–H75, H92), acute respiratory infections (J00–J06, J10–J22), HIV (B20–B24), malignant neoplasms (C69–C97), diseases of the blood and blood forming organs (D55–D89), diabetes mellitus and obesity (E10–E14, E65–E68), mental retardation (F71–F73, F79.1), diseases of the nervous system (G31, G40–G41, G70–G73, G80–G83), heart diseases (I34–I37, I42, I50), diseases of the respiratory system (J35, J40–J47), atopic dermatitis (L20), diseases of the musculoskeletal system and connective tissue (M02–M07, M13, M30–M36), diseases of the kidney (N00–N19), congenital malformations of the circulatory and respiratory system and Down syndrome (Q20–Q39, Q90) and undergone organ transplantations (Z94.0–Z94.6).

In contrast to the NVR and the NIDR, the NRHC does not accumulate in real time and is currently updated once a year. At the time this study was conducted, the NRHC covered patient encounters until the end of 2015, with preliminary data for 2015.

### Statistical analysis

VE was defined as one minus the hazard rate ratio, estimated using Cox regression [12] with the time since the first day of week 40 as underlying time scale. Influenza vaccination was treated as time-dependent variable. VE was estimated for LAIV and TIV separately, using the unvaccinated cohort as a reference for both. Each individual of the study population was followed till the date of LCI, the date of receiving either (i) TIV (when analysing LAIV effectiveness) or (ii) LAIV (when analysing TIV effectiveness), the last day of week 20 or death, whatever occurred first. The validity of the proportional hazards assumption was evaluated using Schoenfeld residuals, and no notable deviation from proportionality was found.

The propensity score method [13] was used to account for potential confounders. In order to include also children with partially missing confounder information, missing values observed in five NMBR variables (Table 1 footnotes d and e; socio-economic status based on mother’s occupation, mother’s marital status, mother’s smoking behaviour, birth weight, gestational age at birth) were imputed using hot deck imputation [14]. Altogether 29 variables, 12 categorical variables derived from NMBR plus one categorical (i.e. number of hospitalisations in 2015, irrespective of the ICD-10 code) and 16 binary variables derived from NRHC, were included into two separate propensity score models estimating each child’s probability of being vaccinated (i) with LAIV and (ii) with TIV conditional on the covariates by applying logistic regression.

The VE estimates were adjusted for (i) LAIV propensity score quintiles in LAIV analysis and (ii) TIV propensity score quintiles in TIV analysis. In addition, further population and outcome subgroup-stratified analyses were conducted according to the child’s seasonal influenza vaccination status in 2013/14 and 2014/15, as well as according to LCI type A and LCI type B.

## Results

### Epidemiology of the 2015/16 influenza season in Finland

The Finnish sentinel surveillance [15] covering a representative sample of all age groups, demonstrated that the influenza season started earlier than usual (in week 47) and spread almost simultaneously all over the country. During the first wave of the season, influenza A(H1N1)pdm09 viruses predominated and all characterized A(H1N1)pdm09 viruses represented the new genetic subclade 6B.1. The second wave was caused by influenza B/Victoria viruses that genetically fell into the B/Brisbane/60/2008 clade. Influenza A(H3N2) viruses belonging to clades 3C.2a and 3C.3a were detected only sporadically. No B/Yamagata viruses were detected in 462 samples tested in the frame of the sentinel surveillance.

### Influenza vaccine effectiveness in two-year-olds

The study population for the VE estimation comprised all permanent residents of Finland eligible for both LAIV and TIV vaccination, i.e. the birth cohort of 2013. Due to small regional and temporal information gaps in the NVR, 5% of the birth cohort 2013 were excluded because of presumably incomplete vaccination records. In addition, 2% that were not found in the NMBR were excluded, leaving 93% of the birth cohort for analysis. The final study population thus comprised 55,258 two-year-old children. The total influenza vaccination coverage was 22%; about two thirds were vaccinated with LAIV and one third with TIV. The characteristics of those included in the analyses are described in Table 1.

Among the 55,258 children, a total 360 LCI were registered in the NIDR. Influenza A cases peaked in week 4 and caused 291 laboratory-confirmed infections. Influenza B mainly circulated between weeks 11 and 14 and caused 69 LCI cases in the study population (Figure). The majority of vaccinations was given before the epidemic (Figure).

**Figure:**
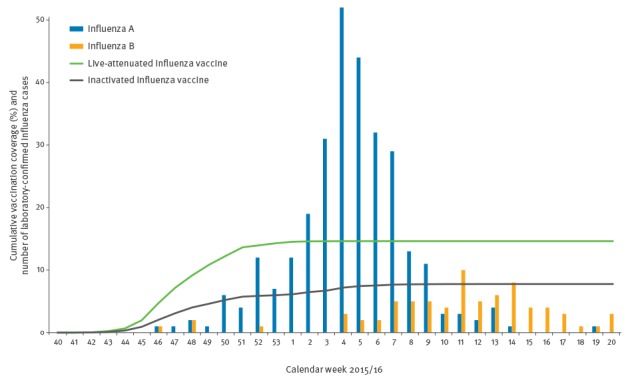
Cumulative seasonal influenza vaccination coverage and number of laboratory-confirmed influenza in two-year-old children by calendar week, Finland, influenza season 2015/16 (n=55,258)

The combined influenza A and B effectiveness estimates adjusted for potential confounders were similar among the LAIV and TIV recipients (51% and 61%, respectively) with widely overlapping confidence intervals (95%CI 28–66 vs. 31–78, respectively), as described in Table 2. The highest effectiveness (80%, 95%CI 50–92) was observed against influenza A among those vaccinated with TIV. Due to small numbers, the influenza B analysis yielded statistically borderline non-significant point estimates (Table 2). The results were practically the same when children were considered vaccinated only after a two-week-period following vaccination (data not shown).

**Table 2:** Influenza vaccine effectiveness against laboratory-confirmed influenza in two-year-old children, stratified by influenza type, Finland, influenza season 2015/16 (n=55,258)^a^

Laboratory-confirmed influenza	Cases			Person-years			Crude effectiveness (95% confidence intervals)		Adjusted effectiveness (95% confidence intervals)	
**Type**	**Not vaccinated**	**LAIV**	**TIV**	**Not vaccinated**	**LAIV**	**TIV**	**LAIV**	**TIV**	**LAIV**	**TIV**
**A and B**	317	31	12	29,984	3,965	1,954	46.5% (22.7%–63.0%)	58.2% (25.6%–76.5%)	50.7% (28.4%–66.1%)	61.2% (30.7%–78.3%)
**A**	260	26	5	29,994	3,967	1,955	45.4% (18.2%–63.5%)	78.2% (47.3%–91.0%)	47.9% (21.6%–65.4%)	79.5% (50.3%–91.6%)
**B**	62	6	7	30,063	3,972	1,957	47.1% (-22.5%–77.1%)	-14.1% (-149.3%–47.8%)	57.2% (-0.0%–81.7%)	-1.0% (-122.8%–54.2%)

LAIV: live attenuated influenza vaccine; TIV: trivalent inactivated influenza vaccine.

^a^ Crude and adjusted for propensity score quintiles.

When stratified by previous exposure to influenza vaccinations, there was a tendency towards higher effectiveness among those previously vaccinated (Table 3), although due to a small number of cases in each stratum, these differences were not statistically significant.

**Table 3:** Influenza vaccine effectiveness against laboratory-confirmed influenza in two-year-old children, stratified by influenza type and seasonal influenza vaccination status in the 2013/14 and 2014/15 seasons, Finland, influenza season 2015/16 (n=55,258)

Laboratory-confirmed influenza		Cases			Person-years			Crude effectiveness (95% confidence intervals)		Adjusted effectiveness (95% confidence intervals)	
**Type**		**Not vaccinated**	**LAIV**	**TIV**	**Not vaccinated**	**LAIV**	**TIV**	**LAIV**	**TIV**	**LAIV**	**TIV**
**A and B**	NPV	272	17	5	25,750	1,691	588	29.3% (-15.4%–56.7%)	40.1% (-45.1%–75.3%)	34.0% (-8.1%–59.7%)	44.1% (-35.7%–76.9%)
	PV	45	14	7	4,234	2,274	1,366	66.2% (38.4%–81.5%)	73.1% (40.4%–87.9%)	69.7% (44.0%–83.6%)	73.3% (40.4%–88.1%)
**A**	NPV	221	15	2	25,759	1,691	589	23.1% (-29.8%–54.4%)	69.3% (-23.4%–92.4%)	24.6% (-27.8%–55.5%)	70.6% (-18.6%–92.7%)
	PV	39	11	3	4,235	2,275	1,367	70.1% (41.6%–84.7%)	86.4% (56.0%–95.8%)	74.0% (48.5%–86.9%)	87.1% (57.9%–96.0%)
**B**	NPV	56	2	3	25,817	1,695	590	60.1% (-63.4%–90.3%)	-51.4% (-383.7%–52.6%)	68.5% (-29.8%–92.4%)	-29.3% (-315.5%–59.8%)
	PV	6	4	4	4,246	2,277	1,367	15.3% (-211.9%–77.0%)	-5.5% (-273.9%–70.2%)	16.7% (-213.7%–77.9%)	-25.1% (-352.0%–65.4%)

LAIV: live attenuated influenza vaccine; NPV: not previously vaccinated; PV: previously vaccinated; TIV: trivalent inactivated influenza vaccine.

^a^ Crude and adjusted for propensity score quintiles.

## Discussion

In Finland, the overall influenza vaccine uptake during the influenza season 2015/16 among two-year-old children was low (22%) but sufficient for a meaningful effectiveness analysis using a nationwide cohort approach. The end-of-season effectiveness estimates were moderately good for both LAIV and TIV with generally slightly higher point estimates for TIV, although the confidence intervals were wide and overlapping. This is in contrast to the findings reported from the US where unlike TIV, LAIV yielded no effectiveness already for the third consecutive season [5]. The LAIV, however, was produced in the same plant for both North American and European markets. The results from the US were based on a test-negative case–control design (TND), and covered children aged 2 to 17 years, in contrast to this study’s cohort design, focusing only on two-year-olds. Our findings are in agreement with those from the UK, where VE in the 2015/16 season was also moderate for influenza A and even good for influenza B [6,16,17] in children and adolescents younger than 18 years and based on a TND.

The particular strength of our study is that by utilising population-based registers, we were able to cover the whole population eligible for LAIV and TIV vaccination; monitoring VE by using routine health registers is particularly suitable for measuring the public health impact of vaccination programmes. Furthermore, the non-preferential national recommendation of influenza vaccinations for two-year-olds for the season 2015/16 allowed us to investigate the effectiveness of LAIV and TIV in parallel within the same cohort.

When using routine registers for defining the exposure, data completeness is a special concern. Therefore the quality and completeness of the NVR is constantly monitored [8] and geographic areas not fulfilling quality criteria are omitted from any cohort analysis. Based on a recent validation study [8] on childhood vaccinations – using MMR vaccination at the age of 12 months as a proxy – the register covers 96% of influenza vaccination records, translating to misclassification of approximately 500 vaccinated in our study cohort. Some LAIV doses may also have been given in the private primary care, which is not currently covered by NVR. However, since all NVP vaccinations are given in public primary care and free of charge, it is anticipated that private primary care uptake in our study cohort was negligible. This is supported by the national pharmaceutical distribution figures in 2015 of 2,120 LAIV doses distributed for the whole eligible age group of 2–17-year-olds. Finally, since lack of data completeness leads to misclassifying a subgroup of those vaccinated to the group of unvaccinated, our VE estimates can be considered conservative, i.e. an underestimation of the real VE.

As with any observational study, the VE estimates may be biased due to unobserved confounders or other types of unknown selection processes in the uptake of vaccinations or care seeking or access to care captured by routine register data. In order to account for potential biases, we adjusted our estimates with several background variables at birth and data of hospital visits prior to the 2015/16 seasonal influenza vaccination campaign. Information on baseline characteristics helps to understand the possible sources of bias in the analysis. The statistically significant differences observed between the three groups, i.e. not vaccinated, LAIV and TIV vaccinated, may not necessarily have clinical significance but underscore the need to perform adjusted analyses. Many of the characteristics thought to increase infection risk, such as siblings, non-Finnish nationality, non-urban residence, low socio-economic status, single mothers and smoking mothers, were more common among the non-vaccinated. Therefore it is somewhat surprising that the adjusted estimates are generally higher than the crude estimates. This may be explained by healthcare-seeking behavior so that parents who get their children vaccinated are possibly also more likely to seek healthcare e.g. for acute respiratory infections like influenza. This is supported by the observation that diagnoses of both chronic and acute diseases prior to the vaccination campaign were more common among the vaccinated. In addition, parents e.g. with higher socio-economic status may predominantly use private primary care, in which the threshold for obtaining laboratory confirmation is presumably lower than in public primary care. Even after adjustment, some residual confounding may still be present.

The role of exposure to previous influenza vaccine doses in the immunological response to subsequent doses has been debated [18]. In young children, two doses have been recommended as necessary for the first time exposure to secure proper priming and maturation of sufficient protection. For LAIV, however, the difference in protection provided by first time one or two doses is marginal [19]. The NVR with vaccination data since year 2009 allows stratified analyses of effectiveness by previously received seasonal influenza vaccine doses; past exposure to influenza vaccines appears to contribute to increased effectiveness in the two-year-old children during the season 2015/16, but due to the relatively small sample size, this difference did not reach statistical significance.

A good antigen match was expected for the quadrivalent LAIV before the start of the 2015/16 influenza epidemic, because the World Health Organization had recommended to change the influenza vaccine composition for both the A(H3N2)- and B-components. Also, the A(H1N1) strain of LAIV was changed due to concerns over its heat instability. Since subtype specific identification of viruses is seldom done in routine clinical practice, our study can reliably address only overall and influenza A VE. The numbers of observations of influenza B viruses were few in this age group and there was not sufficient power to detect VE.

## Conclusion

During the influenza season 2015/16, both LAIV and TIV were effective against laboratory-confirmed influenza among two-year-old children. Finland will continue using LAIV as an alternative intervention to TIV without any official statement on preference. Our study also demonstrates that population-based national health registers are extremely valuable to generate routine data for measuring vaccine impact in a timely manner.

## References

[1] Mooring EQ Bansal S. Increasing herd immunity with influenza revaccination. Epidemiol Infect. 2016;144(6):1267-77. 10.1017/S0950268815002253 26482721

[2] Mereckiene J Cotter S Nicoll A Lopalco P Noori T Weber J VENICE project gatekeepers group. Seasonal influenza immunisation in Europe. Overview of recommendations and vaccination coverage for three seasons: pre-pandemic (2008/09), pandemic (2009/10) and post-pandemic (2010/11). Euro Surveill. 2014;19(16):20780. 10.2807/1560-7917.ES2014.19.16.20780 24786262

[3] Communicable disease act 25.7.1986/583. Available from: http://www.finlex.fi/fi/laki/ajantasa/1986/19860583

[4] Salo H Kilpi T Sintonen H Linna M Peltola V Heikkinen T. Cost-effectiveness of influenza vaccination of healthy children. Vaccine. 2006;24(23):4934-41. 10.1016/j.vaccine.2006.03.057 16678945

[5] Centers for Disease Control and Prevention (CDC). ACIP votes down use of LAIV for 2016-2017 flu season. Updated 22 Jun 2016. Atlanta: CDC; 2016. Available from: http://www.cdc.gov/media/releases/2016/s0622-laiv-flu.html

[6] Pebody R Warburton F Ellis J Andrews N Potts A Cottrell S Effectiveness of seasonal influenza vaccine in preventing laboratory-confirmed influenza in primary care in the United Kingdom: 2015/16 mid-season results. Euro Surveill. 2016;21(13):30179. 10.2807/1560-7917.ES.2016.21.13.30179 27074651

[7] Nohynek H, Baum U, Haveri A, Ikonen N, Jokinen J, Jääskeläinen S, et al. Seasonal childhood influenza vaccinations. Experiences from Finland. Nordic Vaccines Iceland, Apr 2016.

[8] National Institute for Health and Welfare. Finland. Vaccinations. 2016. [Accessed 26 May 2016]. Available from: https://www.thl.fi/fi/web/thlfi-en/statistics/information-on-statistics/quality-descriptions/vaccinations.

[9] Description of the Finnish National Medical Birth Register (NMBR). [Accessed on 19 Sep 2016]. Available from: https://www.thl.fi/fi/web/thlfi-en/statistics/information-on-statistics/register-descriptions/newborns

[10] Sund R. Quality of the Finnish Hospital Discharge Register: a systematic review. Scand J Public Health. 2012;40(6):505-15. 10.1177/1403494812456637 22899561

[11] World Health Organization (WHO). International Statistical Classification of Diseases and Related Health Problems 10th Revision. 2016; Available from: http://apps.who.int/classifications/icd10/browse/2016/en

[12] Cox DR Regression models life-tables. J R Stat Soc B. 1972;34(2):187-220.

[13] Rosenbaum PR Rubin DB The central role of the propensity score in observational studies for causal effect. Biometrika. 1983;70(1):41-55 . 10.1093/biomet/70.1.41

[14] Andridge RR Little RJ. A review of hot deck imputation for survey non-response. Int Stat Rev. 2010;78(1):40-64. 10.1111/j.1751-5823.2010.00103.x 21743766 PMC3130338

[15] Ikonen N, Murtopuro S, Haveri A, Virtanen MJ, Baum U, Nohynek H, et al. Influenssakausi Suomessa, viikot 40/2015–20/2016. THL publications 2016. URN:ISBN:978-952-302-682-7.

[16] Public Health England (PHE). Influenza vaccine effectiveness in adults and children in primary care in the UK: provisional end-of-season results 2015-16. [Accessed 27 Jun 2016]. Available from: https://www.gov.uk/government/uploads/system/uploads/attachment_data/file/530756/Influenza_vaccine_effectiveness_in_primary_care_in_children.pdf

[17] Pebody R Warburton F Ellis J Andrews N Potts A Cottrell S Effectiveness of seasonal influenza vaccine for adults and children in preventing laboratory-confirmed influenza in primary care in the United Kingdom: 2015/16 end-of-season results. Euro Surveill. 2016;21(38):30348 10.2807/1560-7917.ES.2016.21.38.30348 PMC5073201 27684603

[18] Smith DJ Forrest S Ackley DH Perelson AS. Variable efficacy of repeated annual influenza vaccination. Proc Natl Acad Sci USA. 1999;96(24):14001-6. 10.1073/pnas.96.24.14001 10570188 PMC24180

[19] Block SL Toback SL Yi T Ambrose CS. Efficacy of a single dose of live attenuated influenza vaccine in previously unvaccinated children: a post hoc analysis of three studies of children aged 2 to 6 years. Clin Ther. 2009;31(10):2140-7. 10.1016/j.clinthera.2009.09.014 19922885

